# Effective encoder-decoder neural network for segmentation of orbital tissue in computed tomography images of Graves’ orbitopathy patients

**DOI:** 10.1371/journal.pone.0285488

**Published:** 2023-05-10

**Authors:** Seung Hyeun Lee, Sanghyuck Lee, Jaesung Lee, Jeong Kyu Lee, Nam Ju Moon

**Affiliations:** 1 Department of Ophthalmology, Chung-Ang University College of Medicine, Chung-Ang University Hospital, Seoul, Korea; 2 Department of Artificial Intelligence, Chung-Ang University, Seoul, Korea; Cairo University, EGYPT

## Abstract

**Purpose:**

To propose a neural network (NN) that can effectively segment orbital tissue in computed tomography (CT) images of Graves’ orbitopathy (GO) patients.

**Methods:**

We analyzed orbital CT scans from 701 GO patients diagnosed between 2010 and 2019 and devised an effective NN specializing in semantic orbital tissue segmentation in GO patients’ CT images. After four conventional (Attention U-Net, DeepLab V3+, SegNet, and HarDNet-MSEG) and the proposed NN train the various manual orbital tissue segmentations, we calculated the Dice coefficient and Intersection over Union for comparison.

**Results:**

CT images of the eyeball, four rectus muscles, the optic nerve, and the lacrimal gland tissues from all 701 patients were analyzed in this study. In the axial image with the largest eyeball area, the proposed NN achieved the best performance, with Dice coefficients of 98.2% for the eyeball, 94.1% for the optic nerve, 93.0% for the medial rectus muscle, and 91.1% for the lateral rectus muscle. The proposed NN also gave the best performance for the coronal image. Our qualitative analysis demonstrated that the proposed NN outputs provided more sophisticated orbital tissue segmentations for GO patients than the conventional NNs.

**Conclusion:**

We concluded that our proposed NN exhibited an improved CT image segmentation for GO patients over conventional NNs designed for semantic segmentation tasks.

## Introduction

The orbit of the eye is a complex anatomical region, which can be evaluated by a variety of radiological modalities. Computed tomography (CT) is the most widely used diagnostic imaging modality for diagnosing orbital pathologies. Moreover, recent technical advances in the three-dimensional analysis of CT images have enabled the quantitative measure of extraocular muscles, lacrimal glands, and orbital fat [1]. For example, Graves’ orbitopathy (GO) is a well-known orbital disease with extrathyroidal features of dysthyroidism [2]. Using CT to quantitative analyze orbital tissue has become essential for the clinical assessment of GO activity or severity [3]. Currently, however, the manual segmentation of orbital tissues from the CT images remains a labor-intensive and time-consuming process and can be observer-dependent [4, 5].

Previous studies have used neural networks (NNs) to identify, discriminate, and grade various diseases [6–8]. In ophthalmology, NNs are used in the grading of diabetic retinopathy [9], age-related macular degeneration [10], and glaucoma screening [11]. Semantic segmentation of the orbital area using NNs can be a successful application of machine learning techniques because tissues with different Hounsfield units are clustered within a narrow orbit. Recently, several attempts have been made to segment orbital bones, orbital fat, or eye globes in both orbital CT and magnetic resonance imaging using NNs [12–14]. However, due to the architecture of conventional NNs, the restoration performance of the decoder can be limited, which can result in output segments with rough boundaries, particularly when the analyzed CT images contain deformed orbital tissue.

In this study, we propose an effective NN based on encoder-decoder architecture to improve tissue segmentation quality in GO patients. To validate the superiority of the proposed NN, we compare the performance of our proposed NN against four conventional NNs: Attention U-Net [15, 16], DeepLab V3+ [17], SegNet [18], and HarDNet-MSEG [19]. The five NNs are applied to orbital CT images to segment the eyeball, optic nerve, lacrimal gland, and extraocular rectus muscles. We also conduct an in-depth analysis by comparing the segmentation results given by the proposed NN and conventional NNs.

## Materials and methods

This study is a retrospective comparative effectiveness research study. The protocol was approved by the institutional review board of the Chung-Ang University Hospital (IRB No. 2112-013-19395) and complies with the tenets of the Declaration of Helsinki. The requirement for informed consent was waived by the institutional review board because of the retrospective nature of the study.

### Participants

We obtained the orbital CT images (Philips Brilliance 256 Slice iCT, Philips Healthcare Systems, Andover, MA, USA) from 701 GO patients diagnosed between January 2010 and October 2019. Continuous axial scanning was performed with the patient’s head positioned parallel to the Frankfort plane while looking at a fixed point. The scanning parameters were 120 kV, 150 mAs, 64 x 0.625 mm detector configuration, 1.0 mm slice thickness, and 1.0 mm slice increment. Patients with orbital tumors, orbital bone fractures, other orbital structural deformities, or ocular muscle surgery histories were excluded from the study.

### Image acquisition and manual segmentation

Two axial slices and one coronal slice from the respective CT images were selected for each subject. The axial slice displaying the largest eyeball volume was selected and designated as Axial 1; the axial slice expressing the largest lacrimal gland amount was selected and designated as Axial 2. The coronal slice image exhibiting the area located halfway between the eyeball-optic nerve junction and the inner exit of the optic nerve within the orbit was selected and designated as Coronal.

**Fig 1:**
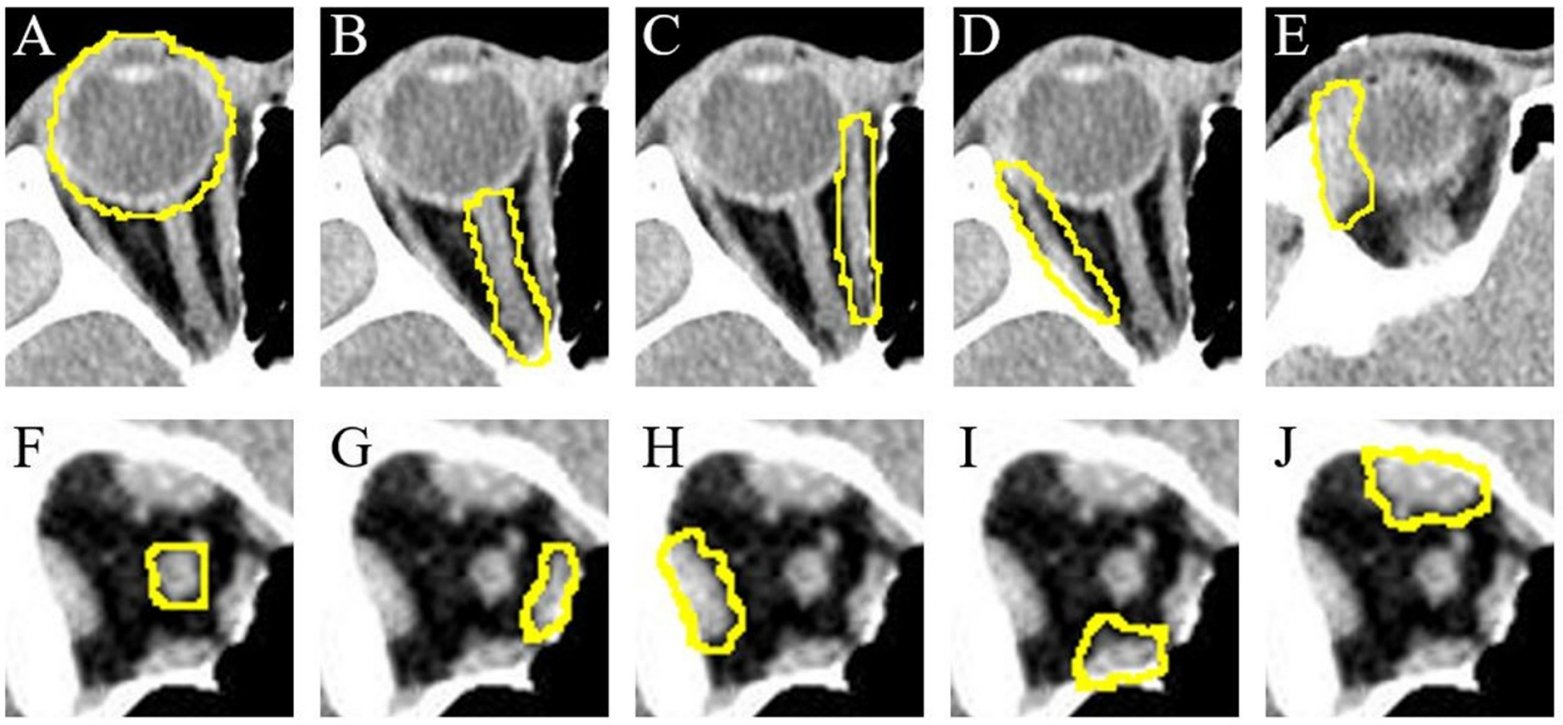
Manual orbital tissue segmentation from (A–E) axial and (F–J) coronal cuts. (A) eyeball, (B) optic nerve, (C) medial rectus muscle, (D) lateral rectus muscle, (E) lacrimal gland, (F) optic nerve, (G) medial rectus muscle, (H) lateral rectus muscle, (I) inferior rectus muscle, and (J) superior rectus muscle.

The boundaries of the eyeball, optic nerve, medial rectus muscle, and lateral rectus muscle are outlined in the Axial 1 image, and the boundary of the lacrimal gland is outlined in the Axial 2 image. The boundaries of the optic nerve, medial, lateral, superior, and inferior rectus muscles are outlined in the Coronal image (Fig 1). Manual segmentation was performed by a single observer using ImageJ software ver. 1.46 (National Institutes of Health, Bethesda, MD, USA; http://rsbweb.nih.gov/ij/).

### Proposed and conventional neural networks

**Fig 2:**
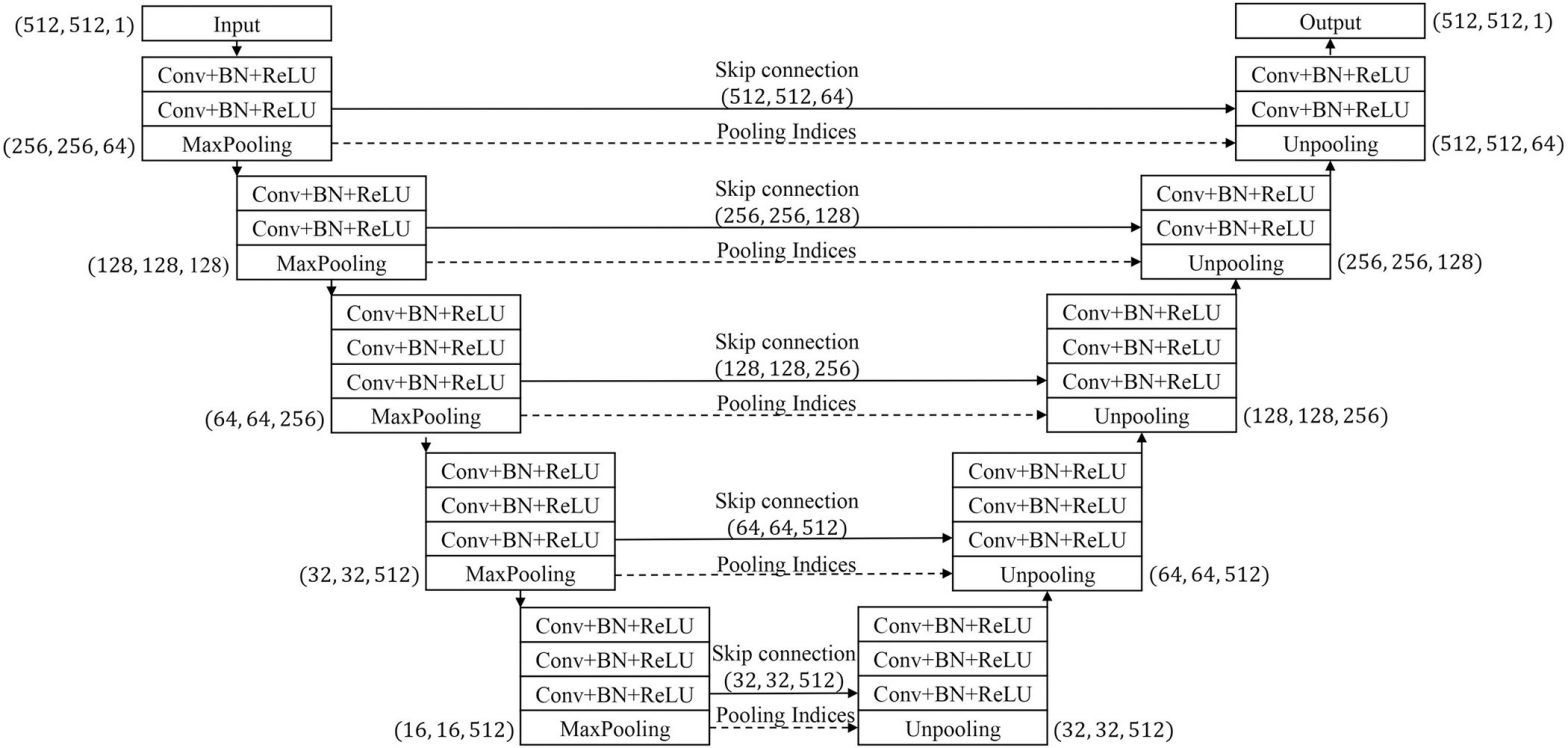
Proposed neural network architecture based on convolution (Conv), batch normalization (BN), rectified linear unit (ReLU), and max pooling operation.

A Fully Convolutional Network (FCN) is a base NN with an architecture that can influence additional NNs for semantic segmentation [20], as FCN variations share a similar architecture with some modifications. For example, U-Net [21], one of the most successful NNs in medical image segmentation, is a modified version of an FCN that strengthens the symmetricity of encoder-decoder architecture. Motivated by this point, we devised our NN for the semantic orbital tissue segmentation in GO patients’ CT images based on the encoder-decoder architecture (Fig 2).

The goal of a five-block encoder is to extract features that contain important information, such as orbital tissue location and boundary. The first two and last three blocks contain two and three convolution layers, respectively. The convolution layer sequentially performs convolution, batch normalization, and rectified linear unit operations. After the convolution layer, one max pooling operation filters out unnecessary information. Because of convolution and max pooling operations, the image size reduces gradually as the original image passes through all five blocks.

Extracted features of each block encompass the image’s varying pixel numbers or area size. For example, the features extracted from the first block cover a small area of the original image; hence, these lowest-level features can help capture the detailed boundary of orbital tissues with complicated shapes, such as the optic nerve. In contrast, the highest-level features extracted from the last block cover a large area of the original image. They can locate the orbital tissue in the image and resist the local noise caused by any deformities. Therefore, low-level features can achieve high-quality tissue segmentation and refine the segmentation boundary by starting from where the highest-level features indicate. In addition, since each block extracts features from the reduced image, the decoder can be designed with counterpart blocks against each encoder block to ensure the compatibility of feature coverage.

The five-block decoder has symmetrical architecture to the encoder, but the max pooling layer of the encoder is replaced with an un-pooling layer for restoring the reduced image to its original size [18]. Initially, the decoder applies an un-pooling operation to the highest-level features delivered from the last block of the encoder. Because up-pooling operation does not require any additional parameters to be trained, more refined values for other trainable parameters can be obtained within a limited number of training epochs. Based on the max pooling indexes used in the pooling layer of the counterpart block in the encoder, image size can be restored by duplicating the feature value to the corresponding location and padding the remaining locations to zero. Next, the features extracted from the counterpart blocks of the encoder are delivered through the skip connection to refine the rough boundary generated through the un-pooling process or alleviate the original boundary’s information loss from the encoding process [21]. These processes are repeated five times until the input image is restored to its original size. As a result, the proposed NN maintains multi-level information to improve the segmentation quality of the orbital tissue of GO patients. The source code of the proposed NN is available at https://github.com/tkdgur658/OTSNet.

This study used four NN semantic segmentation types to verify the proposed NN’s performance: Attention U-Net, DeepLab V3+, SegNet, and HarDNet-MSEG. Attention U-Net, DeepLab V3+, and SegNet are the medical field’s most widely used FCN variants, and HarDNet-MSEG is a medical image segmentation model equipped with the latest NN design techniques. Attention U-Net is a recently improved U-Net variant [22–24] and the most widely used model for medical image segmentation tasks, including orbital structure segmentation. DeepLab V3+, the latest version in the DeepLab series [17, 25, 26], is frequently used for medical image segmentation because of the Atrous convolution’s effectiveness [27–30]. DeepLab V3+ delivers fewer extracted feature types from the encoder than the proposed NN, resulting in a rough segmentation boundary from information loss. Although SegNet was initially developed for road scene images, many variants are certified for medical imaging [31–34]. We used the parameter settings for SegNet suggested by Chandra et al. [34]. Contrasting the proposed NN, SegNet does not exploit additional encoder information except for max pooling indexes during the decoding process. HarDNet-MSEG uses HarDNet as its backbone network and incorporates both received field blocks and cascaded partial decoders for segmentation tasks. HarDNet-MSEG demonstrated promising medical image segmentation performance owing to the receiver field block [35, 36], which is why it was chosen as a compared method in this study. However, unlike the proposed NN, HarDNet-MSEG does not pass low-level feature maps to the decoder.

### Experimental settings

**Fig 3:**
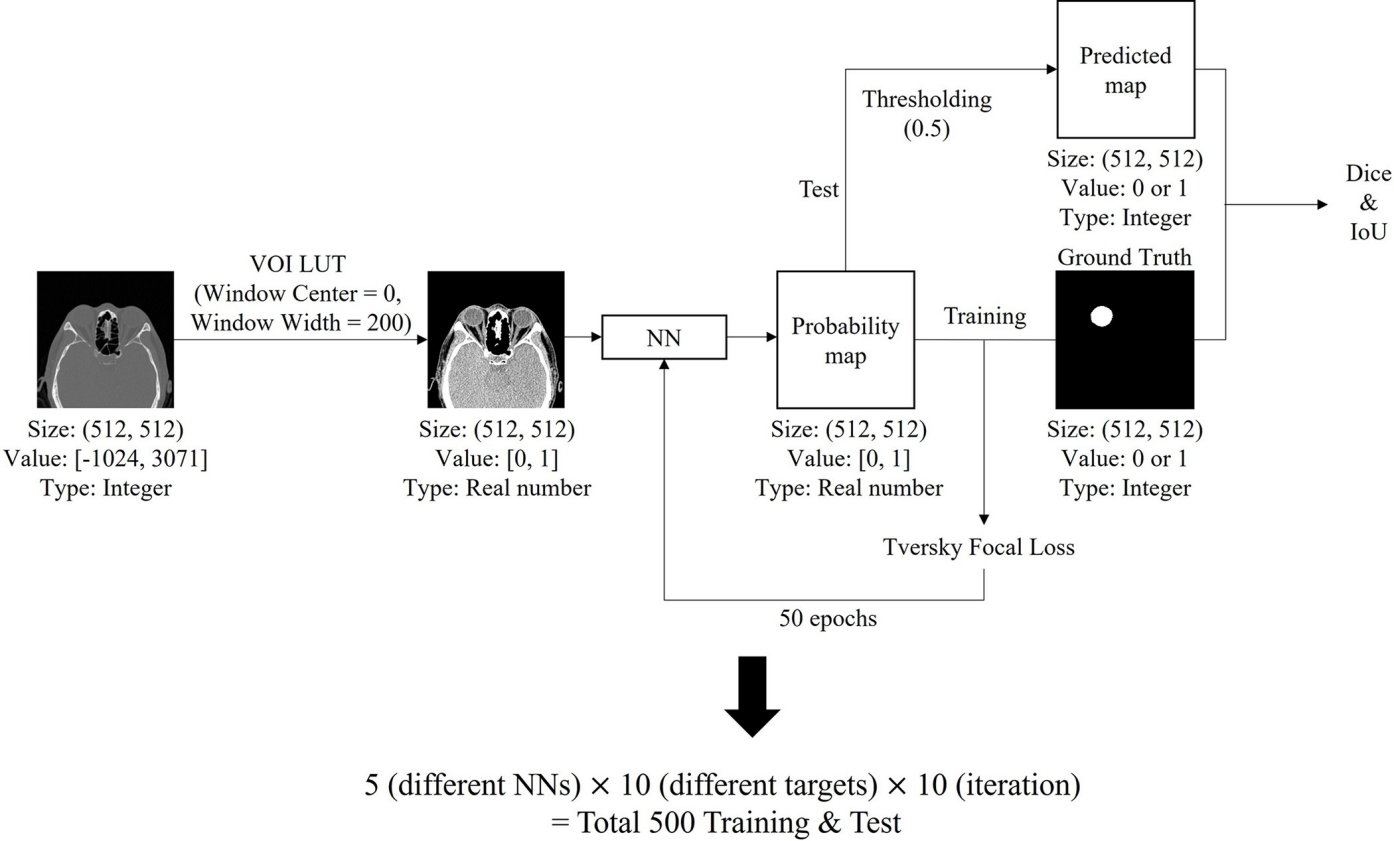
Flowchart of overall training and test process.

Fig 3 illustrates the flowchart of the overall training and test process. The size of the CT images was 512 x 512, and the output was the same size. Before preprocessing, each integer pixel value ranged from -1024 to 3071. The pixels were normalized from 0 to 1 through VOI LUT operation with a Window center of 0 and a Window width of 200. The predicted output value was transformed to 0 for the background and 1 for the orbital tissue by the sigmoid function with a threshold of 0.5. We used Tversky Focal Loss and conducted the tests using a weight with a minimum validation loss value out of 50 epochs. We implemented the five NNs with the Pythorch (1.10.1) library and conducted all experiments in a Geforce RTX 3090 24 GB environment. Hyperparameters comprised batch size, optimizer, learning rate, and weight decay, which were set to 16, AdamW optimizer, 1e-3, and 1e-4, respectively.

### Evaluation

Manually segmented images were used as the ground truth to compare training and performance with the results of the five NNs. For training and evaluation, we randomly split the 701 datasets into training, validation, and test sets with a ratio of 0.7 (training) to 0.15 (validation) to 0.15 (test). Training test sets were repeated ten times for statistic calculations. Overall segmentation performances were evaluated using the Dice coefficient and Intersection over Union (IoU).


$$Dice=\frac{2\times TP}{2\times TP+FP+FN}$$

$$IoU=\frac{TP}{TP+FP+FN}$$

This evaluation suggests that both positive and negative predictive powers are accurate. In the equations, *TP* indicates the number of true positives, *FP* indicates the number of false positives, and *FN* indicates the number of false negatives.

### Statistical analysis

The Dice coefficient and IoU were compared between the five NNs. The values were represented as the mean value with standard deviations. The four remaining NNs’ value distance from the highest segmentation performance criterion (Dice coefficient and IoU) was statistically analyzed using a paired *t*-test. All statistical analyses were performed using the Python library SciPy (https://www.scipy.org), with a *p-*value of < 0.001 denoting statistical significance.

## Results

The age and sex of the 701 participants are shown in Table 1. The number of female participants (503, 71.8%) was much higher than male participants.

**Table 1:** Characteristics of participants.

Age (years, range)	Sex		Total (n, %)
	Male (n, %)	Female (n, %)	
29 and under	59 (8.4)	164 (23.3)	223 (31.7)
30–39	57 (8.1)	170 (24.3)	227 (32.4)
40–49	47 (6.7)	82 (11.7)	129 (18.4)
50–59	26 (3.7)	60 (8.6)	86 (12.3)
60 and over	9 (1.3)	27 (3.9)	36 (5.2)
Total	198 (28.2)	503 (71.8)	701 (100)

Comparisons of the performance of the five NNs, based on the Dice coefficient and IoU for each of the three images, are shown in Tables 2 and 3. In the Axial 1 and 2 images, all the Dice coefficients and IoU values for the segmentation of the five target tissues, including the eyeball, optic nerve, medial rectus muscle, lateral rectus muscle, and lacrimal gland, were highest in the proposed NN (mean values: 98.2%/96.5%, 94.1%/89.5%, 93.0%/88.1%, 91.1%/86.0%, and 87.2%/78.7%, respectively), followed by Attention U-Net (Table 2). The Dice coefficients and IoU values for the five tissues were lowest in the HarDNet-MSEG. In the Coronal image, the Dice coefficient and IoU value for the segmentation of the optic nerve were highest in the proposed NN, followed by SegNet (Table 3). The Dice coefficients and IoU values for the segmentation of the four rectus muscles were also highest in the proposed NN, followed by Attention U-Net (Table 3).

**Table 2:** Comparison of the performance of semantic segmentation using five neural networks in the Axial 1 and 2 images.

Target	Model	mDice	*p*-value	mIoU	*p*-value
Eyeball (Axial 1)	**Proposed**	**98.2 ± 0.1**		**96.5 ± 0.2**	
	HarDNet-MSEG	96.9 ± 0.5	< 0.001	94.2 ± 0.7	< 0.001
	Attention U-Net	97.5 ± 0.2	< 0.001	95.3 ± 0.4	< 0.001
	DeepLab V3+	97.0 ± 0.3	< 0.001	94.5 ± 0.5	< 0.001
	SegNet	97.8 ± 0.1	< 0.001	95.7 ± 0.2	< 0.001
Optic nerve (Axial 1)	**Proposed**	**94.1 ± 0.6**		**89.5 ± 0.8**	
	HarDNet-MSEG	87.5 ± 2.9	< 0.001	79.0 ± 3.3	< 0.001
	Attention U-Net	93.6 ± 0.7	0.005	88.7 ± 1.0	0.006
	DeepLab V3+	91.4 ± 0.7	< 0.001	84.8 ± 0.9	< 0.001
	SegNet	91.8 ± 0.8	< 0.001	85.4 ± 1,1	< 0.001
MRM (Axial 1)	**Proposed**	**93.0 ± 0.7**		**88.1 ± 0.8**	
	HarDNet-MSEG	87.3 ± 1.0	< 0.001	78.6 ± 1.5	< 0.001
	Attention U-Net	92.6 ± 0.7	0.105	87.7 ± 1.1	0.231
	DeepLab V3+	91.0 ± 0.7	< 0.001	84.7 ± 1.3	< 0.001
	SegNet	90.6 ± 1.0	< 0.001	83.9 ± 1.5	< 0.001
LRM (Axial 1)	**Proposed**	**91.1 ± 1.1**		**86.0 ± 1.2**	
	HarDNet-MSEG	81.1 ± 2.5	< 0.001	71.1 ± 2.8	< 0.001
	Attention U-Net	90.2 ± 1.3	< 0.001	85.0 ± 1.5	0.008
	DeepLab V3+	86.9 ± 0.9	< 0.001	79.2 ± 1.0	< 0.001
	SegNet	88,1 ± 0.9	< 0.001	80.8 ± 0.8	< 0.001
Lacrimal gland (Axial 2)	**Proposed**	**87.2 ± 1.9**		**78.7 ± 2.5**	
	HarDNet-MSEG	79.7 ± 3.7	< 0.001	68.1 ± 4.7	< 0.001
	Attention U-Net	81.3 ± 12.2	0.161	71.8 ± 13.5	0.141
	DeepLab V3+	84.0 ± 1.7	< 0.001	73.8 ± 2.2	< 0.001
	SegNet	82.5 ± 1.2	< 0.001	71.8 ± 1.4	< 0.001

MRM; medial rectus muscle, LRM; lateral rectus muscle, mDice; mean Dice, mIoU; mean Intersection over Union.

**Table 3:** Comparison of the performance of semantic segmentation using five neural networks for the Coronal image.

Target	Model	mDice	*p*-value	mIoU	*p*-value
Optic nerve	**Proposed**	**93.2 ± 0.7**		**88.3 ± 1.0**	
	HarDNet-MSEG	85.6 ± 1.8	< 0.001	75.5 ± 2.4	< 0.001
	Attention U-Net	81.7 ± 25.0	0.193	75.7 ± 24.9	0.157
	DeepLab V3+	89.3 ± 0.9	< 0.001	81.4 ± 1.2	< 0.001
	SegNet	91.2 ± 0.9	0.00004	84.9 ± 1.2	0.00002
MRM	**Proposed**	**92.5 ± 1.0**		**87.1 ± 1.4**	
	HarDNet-MSEG	80.3 ± 3.1	< 0.001	68.2 ± 3.9	< 0.001
	Attention U-Net	90.2 ± 3.8	0.108	83.5 ± 5.4	0.087
	DeepLab V3+	87.7 ± 1.0	< 0.001	79.1 ± 1.3	< 0.001
	SegNet	89.3 ± 1.2	< 0.001	81.8 ± 1.6	< 0.001
LRM	**Proposed**	**94.9 ± 0.5**		**90.9 ± 0.9**	
	HarDNet-MSEG	85.6 ± 2.7	< 0.001	76.3 ± 3.4	< 0.001
	Attention U-Net	94.1 ± 0.6	0.003	89.5 ± 0.9	0.00164
	DeepLab V3+	90.6 ± 2.3	< 0.001	83.8 ± 2.2	< 0.001
	SegNet	92.6 ±0.5	< 0.001	86.7 ± 0.9	< 0.001
SRM	**Proposed**	**93.3** ± 0.9		**88.2 ± 1.3**	
	HarDNet-MSEG	85.1 ± 1.6	< 0.001	74.9 ± 2.3	< 0.001
	Attention U-Net	92.2 ± 0.9	0.030	86.2 ± 1.4	0.017
	DeepLab V3+	90.3 ± 0.8	< 0.001	83.0 ± 1.1	< 0.001
	SegNet	90.7 ± 0.9	< 0.001	83.7 ± 1.5	< 0.001
IRM	**Proposed**	**94.5 ± 0.6**		**90.1 ± 0,8**	
	HarDNet-MSEG	83.9 ± 1.9	< 0.001	73.2 ± 2.4	< 0.001
	Attention U-Net	92.4 ± 1.7	0.004	86.8 ± 2.6	0.004
	DeepLab V3+	89.6 ± 0.6	< 0.001	81.7 ± 0.8	< 0.001
	SegNet	91.4 ± 1.4	< 0.001	85.2 ± 2.0	< 0.001

mDice; mean Dice, mIoU; mean Intersection over Union, MRM; medial rectus muscle, LRM; lateral rectus muscle, SRM; superior rectus muscle, IRM; Inferior rectus muscle.

**Fig 4:**
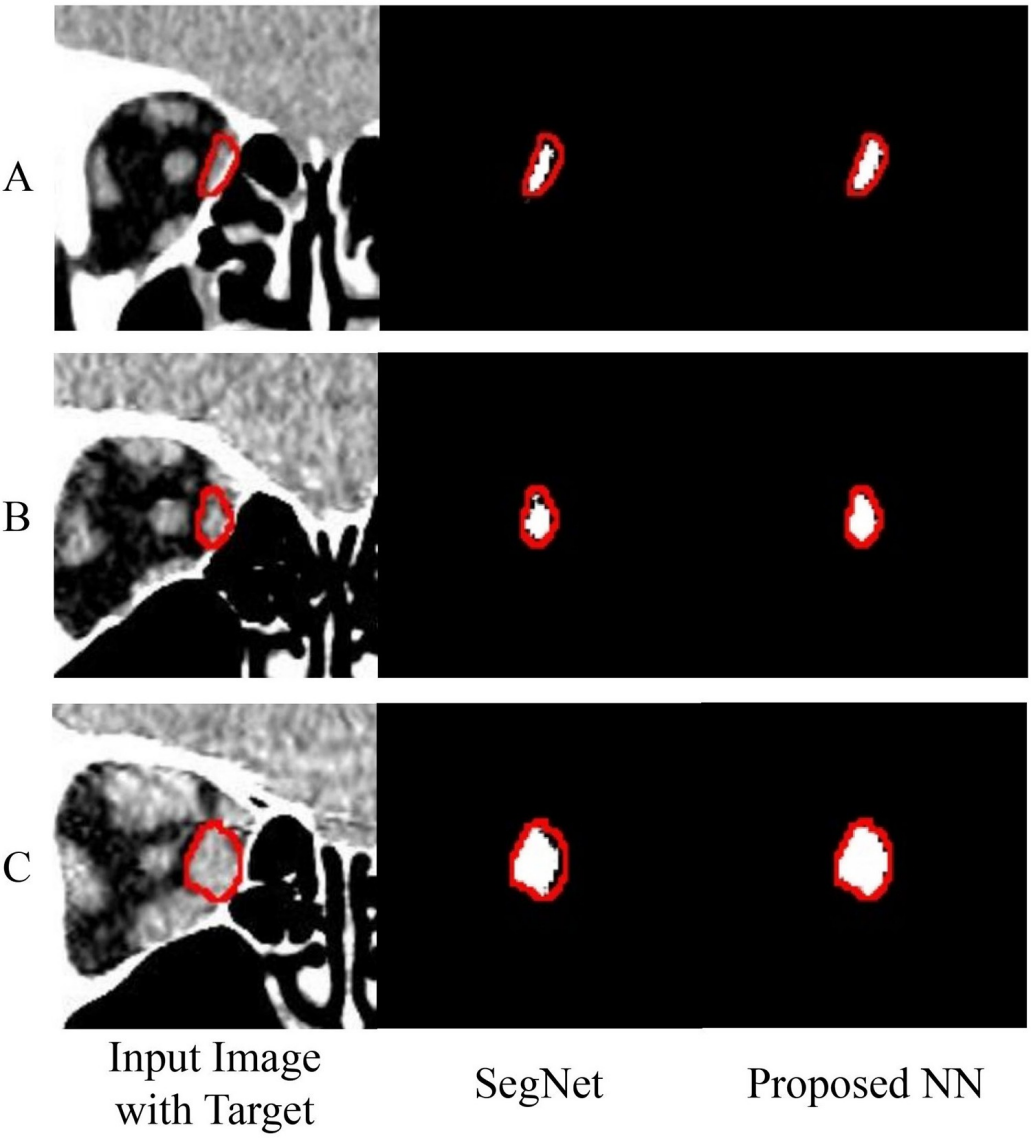
Comparison results between the proposed NN and SegNet. (A) The original images with the boundary of the target tissue are drawn. The visualized segmentation results show the details of the segmentation boundary of the medial rectus muscle given by SegNet (B) and the proposed NN (C), respectively. We visualized the three test images with the largest performance difference in the first experiment iteration.

We conducted a qualitative analysis to demonstrate the superiority of our proposed NN over conventional NNs. We chose SegNet as the counterpart of the proposed NN because of its effectiveness and simple architecture. Fig 4 shows the comparison results between the proposed NN and SegNet. The figure’s first column indicates the input images with the target orbital tissue and the ground truth boundary. The second and third columns signify the segmentation results of SegNet and the proposed NN with white pixels in the ground truth boundary. Thus, perfect orbital tissue segmentation can be confirmed if the ground truth region is fully filled with white pixels. Our results indicated that the proposed NN provides better segmentation results than SegNet, possibly because SegNet does not exploit the multi-level features extracted from the encoding process, except for the max pooling indexes, and also fails to produce sophisticated orbital tissue boundaries.

## Discussion

Several studies have attempted to segment and measure various orbital component areas and volumes using artificial intelligence in orbital CT images (Table 4) [13, 14, 37–41]. However, the model best suited for semantic orbital tissue segmentation in GO patients’ CT images remains unknown. Therefore, it is necessary to determine what NN characteristics are suitable for semantic segmentation. Finding a proper network for semantic segmentation is not only applicable for orbital CT images but essential for supervised learning in many fields of ophthalmology. For example, NNs have been used to segment intraretinal fluid cysts or subretinal fluid on retinal images [42]. Concepts discussed in this study may also apply to other fields of ophthalmology if developed further. NN application in ophthalmology is currently nascent but has many potential clinical uses.

**Table 4:** Literature review of segmentation using artificial intelligence in orbital CT images.

Authors	Study purpose	Dataset	Performance	Limitation
Umapathy et al. (2020)	Globe segmentation for volume quantification	80 subjects	Dice = 0.95	Limited globe injury or contour distortion detection
Hamwood et al. (2021)	Bony orbit segmentation	9 subjects	Dice = 0.813 (orbit) and 0.975 (boundary)	Not feasible for orbital entrance or fractured orbit segmentation
Fu et al. (2021)	Orbital abscess segmentation	67 subjects	Dice = 0.78 Jaccard = 0.65	Image augmentation implemented during training, small dataset
Zhu F et al. (2021)	Total extraocular muscle and optic nerve segmentation	97 subjects	Overall IoU = 0.827	Small dataset, no fixed slice thickness
Li et al. (2022)	Bony orbit segmentation for aging characteristics analysis	595 subjects	Dice = 0.9755	Uses skull CT, not orbital CT
Pan et al (2022)	Bony orbit segmentation for aging parameters	595 subjects	IoU = 0.954	Uses facial skull CT, not orbital CT
Shanker et al. (2022)	Extraocular muscle segmentation	210 subjects	Dice = 0.92 IoU = 0.87	Only uses coronal imaging

One study proposed a predictive tool for reliable segmentation for patient-specific orbital reconstruction in blow-out fractures. The mean Dice coefficient was 88.1% for the automated segmentation of orbital volume using CT scans compared with manual segmentation [43]. Another study attempted three-dimensional reconstruction by automatically segmenting the extraocular muscle and the optic nerve, reporting an 82.1% IoU [38]. In our study, the proposed NN performed well for the various CT planes, with at least an 87.2% Dice coefficient. These results were similar to those of comparable studies and have shown the potential for NNs to replace manual segmentation.

In this study, semantic segmentation of the eyeball showed a high level of accuracy compared with other tissues in CT axial images, probably due to the high contrast of the Hounsfield units for the vitreous cavity and the surrounding fats in the CT images. Since the eyeball contour was always automatically drawn well, developing three-dimensional images of the eyeball using CT imaging will soon be possible. Lacrimal gland size can be affected by various lacrimal tumors and inflammatory conditions, such as GO or pseudotumors [44]. However, there have been no reports of semantic lacrimal gland segmentation using CT images. The Dice coefficient of the lacrimal gland was 87.2% with the proposed NN, which was lower than that of other tissues. This may be caused by the limitations of the CT images, as the lacrimal gland is indistinguishable from the eyeball and periocular tissues. Nevertheless, the resulting accuracy could be applicable in clinical practice.

We established the possibility for diverse semantic orbital tissue segmentation using a NN exhibiting a high agreement level with manual segmentation. Specifically, we devised a NN for semantic segmentation by referencing the architecture and operations to help obtain sophisticated orbital tissue segments in CT images potentially deformed due to GO. Our proposed NN differed from conventional NNs as it specializes in semantically segmenting orbital tissues in GO patients’ CT images. For example, considering multi-level feature exploitation, Attention U-Net’s highest-level features are lower than the proposed NN. Attention U-Net’s minimum feature map is 1/8 of the input size, whereas the proposed NN is 1/16. This difference results from convolution block numbers, including multiple convolutions and poling layers. Therefore, the proposed NN is more efficient regarding orbital tissue size changes due to GO by adding high-level information to the decoder. Similarly, DeepLab V3+, SegNet, and HarDNet-MSEG deliver features extracted from 1/4 and 1/8, with no information, and 1/4, 1/8, and 1/16 of the input size, respectively. In contrast, the proposed NN delivers 1, 1/2, 1/4, 1/8, and 1/16 of the input image to the decoder through the skip connection. DeepLab V3+ and HarDNet-MSEG ignore delivering low-level features to the decoder. Concerning SegNet, the multi-level features’ information loss is intense because it adopts the pooling indexes approach, which is covered qualitatively in Fig 4. This encoder’s strong exploitation power complements the un-pooling technique simplicity, remedying parameter turning difficulties, and improving the segmentation performance. Consequently, the proposed NN outperformed conventional NNs and could reduce the time and effort required for complex manual segmentation. Moreover, the proposed NN (34 million) has a slightly larger parameter size than SegNet (29 million) due to its multi-level skip connection. However, while they have similar architecture, the proposed NN still has a minimal parameter size compared to DeepLabV3+ (59 million).

Although we could investigate orbital tissue segmentation performance using various NNs in multiple CT planes, there were some limitations. First, the manual segmentation of the images was confirmed by a single ophthalmologist and was taken as ground truth. For more reliable semantic segmentation, multiple specialists should be consulted regarding the segmentation of orbital tissues to reduce segmentation errors. Since CNN-based deep learning requires considerable data, a larger dataset can further improve segmentation performance. Additionally, there was potential bias in the manual axial and coronal cuts representation choice. For example, the Axial 2 image is a slice of the maximum lacrimal gland area. However, the selection of the slice could differ among individuals. Considering technology, the proposed NN’s naïve skip connection use may be a possible limitation. Existing works have proposed various skip connection approaches, such as convolution in skip connection and connecting multi-level features from multiple encoders to one decoder. Thus, future research should focus on multi-level feature use in decoding, and the detailed design for their effective combination must be investigated more thoroughly.

In conclusion, we introduced an effective encoder-decoder NN of orbital tissue segmentation in GO patients’ CT. The proposed encoder delivers low- and high-level features to the decoder for capturing clear boundaries and concurrently resisting local noise. Then, the decoder exploits these features with an un-pooling operation and skip connection for effective image size restoration. The experimental results indicated that the proposed architecture significantly outperformed four conventional NNs types designed for semantic segmentation. Technically, the proposed model encourages using multi-level decoding features to obtain sophisticated target boundaries potentially deformed due to GO. This study provides a fundamental basis for automatic GO evaluation, which could replace manual CT image evaluation if developed further.

## Supporting information

S1 Appendix (DOCX)
