# Supplementary material for: Effective encoder-decoder neural network for segmentation of orbital tissue in computed tomography images of Graves’ orbitopathy patients
Source: PLoS One. 2023 May 10;18(5):e0285488. doi: 10.1371/journal.pone.0285488 (PMC10171592; doi:10.1371/journal.pone.0285488)
Supplement: S1 Appendix — (DOCX) [file pone.0285488.s001.docx]

**Table S1. Comparison of the performance of eyeball segmentation for the Axial 1 image**

| **Target** | **Iteration** | **Model** | **mDice** | **mIoU** |
| --- | --- | --- | --- | --- |
| Eyeball | 1 | **Proposed** | **98.10%** | **96.3%** |
|  |  | HarDNet-MSEG | 97.20% | 94.6% |
|  |  | Attention U-Net | 97.60% | 95.5% |
|  |  | DeepLab V3+ | 96.90% | 94.1% |
|  |  | SegNet | 97.80% | 95.8% |
|  | 2 | **Proposed** | **98.20%** | **96.6%** |
|  |  | HarDNet-MSEG | 96.60% | 93.9% |
|  |  | Attention U-Net | 97.40% | 95.1% |
|  |  | DeepLab V3+ | 96.70% | 94.3% |
|  |  | SegNet | 97.80% | 95.7% |
|  | 3 | **Proposed** | **98.00%** | **96.2%** |
|  |  | HarDNet-MSEG | 97.00% | 94.2% |
|  |  | Attention U-Net | 97.30% | 94.8% |
|  |  | DeepLab V3+ | 97.20% | 94.7% |
|  |  | SegNet | 97.70% | 95.6% |
|  | 4 | **Proposed** | **98.30%** | **96.6%** |
|  |  | HarDNet-MSEG | 97.00% | 94.2% |
|  |  | Attention U-Net | 97.70% | 95.7% |
|  |  | DeepLab V3+ | 96.30% | 93.5% |
|  |  | SegNet | 97.80% | 95.7% |
|  | 5 | **Proposed** | **98.20%** | **96.5%** |
|  |  | HarDNet-MSEG | 97.00% | 94.4% |
|  |  | Attention U-Net | 97.20% | 94.8% |
|  |  | DeepLab V3+ | 97.30% | 94.8% |
|  |  | SegNet | 97.70% | 95.5% |
|  | 6 | **Proposed** | **98.30%** | **96.8%** |
|  |  | HarDNet-MSEG | 95.70% | 92.6% |
|  |  | Attention U-Net | 97.20% | 94.7% |
|  |  | DeepLab V3+ | 97.20% | 94.6% |
|  |  | SegNet | 97.80% | 95.7% |
|  | 7 | **Proposed** | **98.10%** | **96.3%** |
|  |  | HarDNet-MSEG | 97.30% | 94.9% |
|  |  | Attention U-Net | 97.70% | 95.6% |
|  |  | DeepLab V3+ | 97.20% | 94.7% |
|  |  | SegNet | 97.70% | 95.6% |
|  | 8 | **Proposed** | **98.20%** | **96.6%** |
|  |  | HarDNet-MSEG | 96.40% | 93.3% |
|  |  | Attention U-Net | 97.50% | 95.2% |
|  |  | DeepLab V3+ | 97.20% | 94.6% |
|  |  | SegNet | 97.80% | 95.8% |
|  | 9 | **Proposed** | **98.30%** | **96.7%** |
|  |  | HarDNet-MSEG | 97.50% | 95.2% |
|  |  | Attention U-Net | 97.80% | 95.8% |
|  |  | DeepLab V3+ | 97.60% | 95.3% |
|  |  | SegNet | 98.00% | 96.2% |
|  | 10 | **Proposed** | **98.20%** | **96.4%** |
|  |  | HarDNet-MSEG | 97.10% | 94.3% |
|  |  | Attention U-Net | 97.70% | 95.6% |
|  |  | DeepLab V3+ | 96.80% | 94.4% |
|  |  | SegNet | 97.70% | 95.6% |

mDice; mean Dice, mIoU, mean Intersection over Union.

**Table S2. Comparison of the performance of optic nerve segmentation for the Axial 1 image**

| **Target** | **Iteration** | **Model** | **mDice** | **mIoU** |
| --- | --- | --- | --- | --- |
| Optic nerve | 1 | **Proposed** | **94.30%** | **89.7%** |
|  |  | HarDNet-MSEG | 88.10% | 79.9% |
|  |  | Attention U-Net | 93.10% | 87.8% |
|  |  | DeepLab V3+ | 91.70% | 85.1% |
|  |  | SegNet | 91.60% | 85.0% |
|  | 2 | **Proposed** | **93.80%** | **89.3%** |
|  |  | HarDNet-MSEG | 87.30% | 78.2% |
|  |  | Attention U-Net | 93.70% | **89.3%** |
|  |  | DeepLab V3+ | 91.50% | 85.3% |
|  |  | SegNet | 91.60% | 85.2% |
|  | 3 | **Proposed** | **94.80%** | **90.3%** |
|  |  | HarDNet-MSEG | 90.60% | 83.0% |
|  |  | Attention U-Net | 94.10% | 89.1% |
|  |  | DeepLab V3+ | 92.50% | 86.3% |
|  |  | SegNet | 92.80% | 86.9% |
|  | 4 | **Proposed** | **95.40%** | **91.3%** |
|  |  | HarDNet-MSEG | 88.30% | 79.8% |
|  |  | Attention U-Net | 94.80% | 90.5% |
|  |  | DeepLab V3+ | 92.40% | 86.2% |
|  |  | SegNet | 93.10% | 87.4% |
|  | 5 | **Proposed** | **93.90%** | **89.3%** |
|  |  | HarDNet-MSEG | 87.20% | 78.7% |
|  |  | Attention U-Net | 93.70% | 89.2% |
|  |  | DeepLab V3+ | 90.80% | 84.0% |
|  |  | SegNet | 91.50% | 85.2% |
|  | 6 | **Proposed** | **93.50%** | **89.0%** |
|  |  | HarDNet-MSEG | 86.50% | 77.9% |
|  |  | Attention U-Net | 92.40% | 87.4% |
|  |  | DeepLab V3+ | 91.00% | 84.3% |
|  |  | SegNet | 91.00% | 84.7% |
|  | 7 | **Proposed** | **93.70%** | **89.1%** |
|  |  | HarDNet-MSEG | 89.50% | 81.5% |
|  |  | Attention U-Net | 93.40% | 88.7% |
|  |  | DeepLab V3+ | 90.70% | 83.6% |
|  |  | SegNet | 91.20% | 84.6% |
|  | 8 | **Proposed** | **93.20%** | **88.0%** |
|  |  | HarDNet-MSEG | 87.30% | 78.1% |
|  |  | Attention U-Net | 92.60% | 87.0% |
|  |  | DeepLab V3+ | 89.90% | 83.4% |
|  |  | SegNet | 90.60% | 83.7% |
|  | 9 | **Proposed** | **94.00%** | **89.6%** |
|  |  | HarDNet-MSEG | 90.20% | 82.7% |
|  |  | Attention U-Net | **94.00%** | 89.2% |
|  |  | DeepLab V3+ | 91.40% | 84.8% |
|  |  | SegNet | 92.50% | 86.5% |
|  | 10 | **Proposed** | **93.90%** | **89.1%** |
|  |  | HarDNet-MSEG | 79.80% | 70.7% |
|  |  | Attention U-Net | 93.80% | 89.0% |
|  |  | DeepLab V3+ | 91.60% | 85.0% |
|  |  | SegNet | 91.70% | 85.3% |

mDice; mean Dice, mIoU, mean Intersection over Union.

**Table S3. Comparison of the performance of MRM segmentation for the Axial 1 image**

| **Target** | **Iteration** | **Model** | **mDice** | **mIoU** |
| --- | --- | --- | --- | --- |
| MRM | 1 | Proposed | 93.30% | 88.6% |
|  |  | HarDNet-MSEG | 88.20% | 79.9% |
|  |  | **Attention U-Net** | **93.40%** | **89.0%** |
|  |  | DeepLab V3+ | 91.10% | 84.8% |
|  |  | SegNet | 90.00% | 83.1% |
|  | 2 | **Proposed** | **93.30%** | **88.6%** |
|  |  | HarDNet-MSEG | 88.00% | 79.6% |
|  |  | Attention U-Net | 91.80% | 86.4% |
|  |  | DeepLab V3+ | 91.70% | 85.9% |
|  |  | SegNet | 91.00% | 84.6% |
|  | 3 | **Proposed** | **93.10%** | 87.6% |
|  |  | HarDNet-MSEG | 87.80% | 79.2% |
|  |  | **Attention U-Net** | 93.00% | **87.8%** |
|  |  | DeepLab V3+ | 90.10% | 82.7% |
|  |  | SegNet | 91.80% | 85.5% |
|  | 4 | **Proposed** | **94.40%** | **89.8%** |
|  |  | HarDNet-MSEG | 88.50% | 79.8% |
|  |  | Attention U-Net | 93.70% | 88.8% |
|  |  | DeepLab V3+ | 92.30% | 86.4% |
|  |  | SegNet | 92.10% | 85.9% |
|  | 5 | Proposed | 92.40% | 87.8% |
|  |  | HarDNet-MSEG | 87.00% | 78.7% |
|  |  | **Attention U-Net** | **93.10%** | **89.1%** |
|  |  | DeepLab V3+ | 91.50% | 86.3% |
|  |  | SegNet | 88.60% | 80.6% |
|  | 6 | Proposed | **93.00%** | 88.0% |
|  |  | HarDNet-MSEG | 85.30% | 75.3% |
|  |  | **Attention U-Net** | **93.00%** | **88.1%** |
|  |  | DeepLab V3+ | 89.90% | 82.7% |
|  |  | SegNet | 91.50% | 85.3% |
|  | 7 | Proposed | 91.80% | 86.7% |
|  |  | HarDNet-MSEG | 86.70% | 78.3% |
|  |  | **Attention U-Net** | **92.00%** | **87.1%** |
|  |  | DeepLab V3+ | 90.70% | 84.4% |
|  |  | SegNet | 90.10% | 83.2% |
|  | 8 | **Proposed** | **92.60%** | **87.4%** |
|  |  | HarDNet-MSEG | 87.10% | 78.5% |
|  |  | Attention U-Net | 91.30% | 85.6% |
|  |  | DeepLab V3+ | 90.50% | 83.8% |
|  |  | SegNet | 90.10% | 83.0% |
|  | 9 | **Proposed** | **93.30%** | **88.6%** |
|  |  | HarDNet-MSEG | 88.20% | 79.9% |
|  |  | Attention U-Net | 92.90% | 88.1% |
|  |  | DeepLab V3+ | 90.70% | 84.3% |
|  |  | SegNet | 90.80% | 84.5% |
|  | 10 | **Proposed** | **93.30%** | **88.1%** |
|  |  | HarDNet-MSEG | 86.30% | 76.6% |
|  |  | Attention U-Net | 92.20% | 86.6% |
|  |  | DeepLab V3+ | 91.80% | 85.4% |
|  |  | SegNet | 90.30% | 83.2% |

mDice; mean Dice, mIoU, mean Intersection over Union, MRM; medial rectus muscle.

**Table S4. Comparison of the performance of LRM segmentation for the Axial 1 image**

| **Target** | **Iteration** | **Model** | **mDice** | **mIoU** |
| --- | --- | --- | --- | --- |
| LRM | 1 | **Proposed** | **90.9%** | **85.7%** |
|  |  | HarDNet-MSEG | 80.6% | 70.8% |
|  |  | Attention U-Net | 90.2% | 85.4% |
|  |  | DeepLab V3+ | 87.0% | 79.8% |
|  |  | SegNet | 87.4% | 81.1% |
|  | 2 | **Proposed** | **89.4%** | **84.8%** |
|  |  | HarDNet-MSEG | 77.8% | 66.0% |
|  |  | Attention U-Net | 87.8% | 81.6% |
|  |  | DeepLab V3+ | 85.7% | 78.3% |
|  |  | SegNet | 86.8% | 80.0% |
|  | 3 | **Proposed** | **90.9%** | **84.9%** |
|  |  | HarDNet-MSEG | 83.0% | 72.6% |
|  |  | Attention U-Net | 90.5% | 84.5% |
|  |  | DeepLab V3+ | 86.6% | 78.4% |
|  |  | SegNet | 89.0% | 81.7% |
|  | 4 | **Proposed** | **91.3%** | **86.8%** |
|  |  | HarDNet-MSEG | 82.3% | 72.7% |
|  |  | Attention U-Net | 90.0% | 85.6% |
|  |  | DeepLab V3+ | 86.1% | 78.0% |
|  |  | SegNet | 87.5% | 79.8% |
|  | 5 | **Proposed** | **89.1%** | **84.2%** |
|  |  | HarDNet-MSEG | 78.9% | 68.1% |
|  |  | Attention U-Net | 88.1% | 83.8% |
|  |  | DeepLab V3+ | 85.5% | 78.7% |
|  |  | SegNet | 86.7% | 79.8% |
|  | 6 | **Proposed** | **92.2%** | **87.2%** |
|  |  | HarDNet-MSEG | 78.4% | 70.0% |
|  |  | Attention U-Net | 90.9% | 86.2% |
|  |  | DeepLab V3+ | 87.8% | 80.3% |
|  |  | SegNet | 88.8% | 81.5% |
|  | 7 | **Proposed** | **91.7%** | **86.6%** |
|  |  | HarDNet-MSEG | 79.3% | 70.1% |
|  |  | Attention U-Net | 91.0% | 85.6% |
|  |  | DeepLab V3+ | 87.8% | 80.1% |
|  |  | SegNet | 88.5% | 80.9% |
|  | 8 | **Proposed** | **90.7%** | **84.6%** |
|  |  | HarDNet-MSEG | 80.9% | 70.4% |
|  |  | Attention U-Net | 90.1% | 84.2% |
|  |  | DeepLab V3+ | 86.4% | 77.8% |
|  |  | SegNet | 87.9% | 80.4% |
|  | 9 | **Proposed** | **92.1%** | **87.0%** |
|  |  | HarDNet-MSEG | 83.6% | 74.1% |
|  |  | Attention U-Net | 91.2% | 86.7% |
|  |  | DeepLab V3+ | 87.7% | 80.5% |
|  |  | SegNet | 88.4% | 81.0% |
|  | 10 | **Proposed** | **92.9%** | **87.7%** |
|  |  | HarDNet-MSEG | 86.2% | 76.4% |
|  |  | Attention U-Net | 92.1% | 86.6% |
|  |  | DeepLab V3+ | 88.2% | 79.6% |
|  |  | SegNet | 89.6% | 82.2% |

mDice; mean Dice, mIoU, mean Intersection over Union, LRM; lateral rectus muscle.

**Table S5. Comparison of the performance of larcrimal gland segmentation for the Axial 2 image**

| **Target** | **Iteration** | **Model** | **mDice** | **mIoU** |
| --- | --- | --- | --- | --- |
| Lacrimal gland | 1 | **Proposed** | **82.2%** | **71.8%** |
|  |  | HarDNet-MSEG | 75.3% | 63.9% |
|  |  | Attention U-Net | 77.1% | 65.6% |
|  |  | DeepLab V3+ | 80.7% | 69.9% |
|  |  | SegNet | 79.8% | 69.1% |
|  | 2 | **Proposed** | **87.3%** | **79.1%** |
|  |  | HarDNet-MSEG | 83.0% | 72.7% |
|  |  | Attention U-Net | 86.4% | 78.1% |
|  |  | DeepLab V3+ | 85.4% | 75.9% |
|  |  | SegNet | 82.6% | 72.3% |
|  | 3 | **Proposed** | **86.9%** | **78.0%** |
|  |  | HarDNet-MSEG | 79.7% | 67.4% |
|  |  | Attention U-Net | 85.3% | 75.7% |
|  |  | DeepLab V3+ | 82.6% | 71.4% |
|  |  | SegNet | 82.5% | 71.4% |
|  | 4 | **Proposed** | **88.4%** | **80.2%** |
|  |  | HarDNet-MSEG | 86.3% | 76.5% |
|  |  | Attention U-Net | 87.5% | 79.0% |
|  |  | DeepLab V3+ | 83.4% | 73.1% |
|  |  | SegNet | 84.0% | 73.5% |
|  | 5 | **Proposed** | **86.1%** | **77.7%** |
|  |  | HarDNet-MSEG | 74.1% | 61.5% |
|  |  | Attention U-Net | 46.1% | 33.2% |
|  |  | DeepLab V3+ | 83.2% | 73.1% |
|  |  | SegNet | 81.8% | 71.2% |
|  | 6 | **Proposed** | **87.9%** | 79.5% |
|  |  | HarDNet-MSEG | 82.6% | 71.7% |
|  |  | **Attention U-Net** | 87.4% | **79.6%** |
|  |  | DeepLab V3+ | 83.6% | 72.6% |
|  |  | SegNet | 82.6% | 71.6% |
|  | 7 | Proposed | 88.4% | 79.8% |
|  |  | HarDNet-MSEG | 75.4% | 61.6% |
|  |  | **Attention U-Net** | **89.5%** | **81.5%** |
|  |  | DeepLab V3+ | 86.9% | 77.4% |
|  |  | SegNet | 82.9% | 71.6% |
|  | 8 | **Proposed** | **88.1%** | **79.9%** |
|  |  | HarDNet-MSEG | 78.8% | 66.1% |
|  |  | Attention U-Net | 85.0% | 75.7% |
|  |  | DeepLab V3+ | 86.0% | 76.5% |
|  |  | SegNet | 81.5% | 70.2% |
|  | 9 | **Proposed** | **87.0%** | **79.0%** |
|  |  | HarDNet-MSEG | 81.2% | 70.5% |
|  |  | Attention U-Net | 83.1% | 73.0% |
|  |  | DeepLab V3+ | 83.6% | 73.6% |
|  |  | SegNet | 83.7% | 73.6% |
|  | 10 | **Proposed** | **89.3%** | **81.6%** |
|  |  | HarDNet-MSEG | 80.8% | 69.1% |
|  |  | Attention U-Net | 85.6% | 76.3% |
|  |  | DeepLab V3+ | 84.5% | 74.2% |
|  |  | SegNet | 83.6% | 73.3% |

mDice; mean Dice, mIoU, mean Intersection over Union.

**Table S6. Comparison of the performance of optic nerve segmentation for the Coronal image**

| **Target** | **Iteration** | **Model** | **mDice** | **mIoU** |
| --- | --- | --- | --- | --- |
| Optic nerve | 1 | **Proposed** | **93.2%** | **87.9%** |
|  |  | HarDNet-MSEG | 83.9% | 72.8% |
|  |  | Attention U-Net | 92.9% | 87.7% |
|  |  | DeepLab V3+ | 89.5% | 81.6% |
|  |  | SegNet | 90.6% | 84.0% |
|  | 2 | **Proposed** | **93.3%** | **88.1%** |
|  |  | HarDNet-MSEG | 87.4% | 78.0% |
|  |  | Attention U-Net | 91.0% | 84.6% |
|  |  | DeepLab V3+ | 89.7% | 82.3% |
|  |  | SegNet | 89.6% | 82.5% |
|  | 3 | **Proposed** | **94.0%** | **89.2%** |
|  |  | HarDNet-MSEG | 87.5% | 78.1% |
|  |  | **Attention U-Net** | **94.0%** | **89.2%** |
|  |  | DeepLab V3+ | 89.9% | 81.9% |
|  |  | SegNet | 91.3% | 84.5% |
|  | 4 | **Proposed** | **93.6%** | 88.6% |
|  |  | HarDNet-MSEG | 86.8% | 77.2% |
|  |  | **Attention U-Net** | 93.1% | **88.7%** |
|  |  | DeepLab V3+ | 90.5% | 83.1% |
|  |  | SegNet | 92.6% | 86.8% |
|  | 5 | **Proposed** | **92.6%** | **86.8%** |
|  |  | HarDNet-MSEG | 87.6% | 78.3% |
|  |  | Attention U-Net | 90.0% | 82.8% |
|  |  | DeepLab V3+ | 89.1% | 81.0% |
|  |  | SegNet | 91.7% | 85.4% |
|  | 6 | **Proposed** | **92.0%** | **86.9%** |
|  |  | HarDNet-MSEG | 85.4% | 75.5% |
|  |  | Attention U-Net | 9.3% | 5.7% |
|  |  | DeepLab V3+ | 87.5% | 78.8% |
|  |  | SegNet | 90.5% | 84.5% |
|  | 7 | **Proposed** | 92.5% | 88.1% |
|  |  | HarDNet-MSEG | 84.2% | 74.2% |
|  |  | Attention U-Net | **93.3%** | **88.5%** |
|  |  | DeepLab V3+ | 89.0% | 81.1% |
|  |  | SegNet | 91.0% | 84.9% |
|  | 8 | **Proposed** | **93.5%** | **89.1%** |
|  |  | HarDNet-MSEG | 84.7% | 74.5% |
|  |  | Attention U-Net | 70.6% | 58.5% |
|  |  | DeepLab V3+ | 90.2% | 82.5% |
|  |  | SegNet | 91.8% | 85.9% |
|  | 9 | **Proposed** | **94.7%** | **90.3%** |
|  |  | HarDNet-MSEG | 86.8% | 77.4% |
|  |  | Attention U-Net | 92.8% | 87.4% |
|  |  | DeepLab V3+ | 89.4% | 81.4% |
|  |  | SegNet | 92.6% | 86.6% |
|  | 10 | **Proposed** | **93.0%** | **88.1%** |
|  |  | HarDNet-MSEG | 82.0% | 70.9% |
|  |  | Attention U-Net | 89.9% | 84.0% |
|  |  | DeepLab V3+ | 88.1% | 80.1% |
|  |  | SegNet | 90.8% | 84.1% |

mDice; mean Dice, mIoU, mean Intersection over Union.

**Table S7. Comparison of the performance of MRM segmentation for the Coronal image**

| **Target** | **Iteration** | **Model** | **mDice** | **mIoU** |
| --- | --- | --- | --- | --- |
| MRM | 1 | **Proposed** | **92.4%** | **86.6%** |
|  |  | HarDNet-MSEG | 76.2% | 62.7% |
|  |  | Attention U-Net | 90.3% | 83.6% |
|  |  | DeepLab V3+ | 88.9% | 80.7% |
|  |  | SegNet | 89.2% | 81.3% |
|  | 2 | Proposed | 91.2% | 84.8% |
|  |  | HarDNet-MSEG | 82.3% | 70.5% |
|  |  | **Attention U-Net** | **91.3%** | **85.0%** |
|  |  | DeepLab V3+ | 86.1% | 77.5% |
|  |  | SegNet | 89.3% | 81.7% |
|  | 3 | **Proposed** | **93.7%** | **88.8%** |
|  |  | HarDNet-MSEG | 82.2% | 70.2% |
|  |  | Attention U-Net | 92.0% | 85.9% |
|  |  | DeepLab V3+ | 88.9% | 80.6% |
|  |  | SegNet | 91.2% | 84.5% |
|  | 4 | **Proposed** | **93.7%** | **88.8%** |
|  |  | HarDNet-MSEG | 82.1% | 70.2% |
|  |  | Attention U-Net | 91.7% | 85.4% |
|  |  | DeepLab V3+ | 87.7% | 79.1% |
|  |  | SegNet | 89.1% | 81.4% |
|  | 5 | Proposed | 91.3% | 85.5% |
|  |  | HarDNet-MSEG | 79.0% | 66.4% |
|  |  | **Attention U-Net** | **91.9%** | **85.8%** |
|  |  | DeepLab V3+ | 87.7% | 78.8% |
|  |  | SegNet | 87.2% | 78.8% |
|  | 6 | **Proposed** | **92.5%** | **86.8%** |
|  |  | HarDNet-MSEG | 81.1% | 68.7% |
|  |  | Attention U-Net | 78.9% | 67.5% |
|  |  | DeepLab V3+ | 88.9% | 80.5% |
|  |  | SegNet | 89.1% | 81.2% |
|  | 7 | **Proposed** | **92.7%** | **87.2%** |
|  |  | HarDNet-MSEG | 80.4% | 67.8% |
|  |  | Attention U-Net | 91.9% | 86.1% |
|  |  | DeepLab V3+ | 87.7% | 78.8% |
|  |  | SegNet | 89.9% | 82.8% |
|  | 8 | **Proposed** | 92.3% | **87.2%** |
|  |  | HarDNet-MSEG | 76.0% | 63.3% |
|  |  | **Attention U-Net** | **92.6%** | 87.1% |
|  |  | DeepLab V3+ | 86.6% | 77.6% |
|  |  | SegNet | 90.0% | 83.0% |
|  | 9 | **Proposed** | **94.0%** | **89.0%** |
|  |  | HarDNet-MSEG | 86.5% | 76.5% |
|  |  | Attention U-Net | 91.0% | 84.3% |
|  |  | DeepLab V3+ | 88.4% | 80.1% |
|  |  | SegNet | 90.6% | 83.6% |
|  | 10 | **Proposed** | **91.6%** | **85.9%** |
|  |  | HarDNet-MSEG | 77.3% | 65.3% |
|  |  | Attention U-Net | 90.5% | 84.7% |
|  |  | DeepLab V3+ | 86.3% | 77.4% |
|  |  | SegNet | 87.5% | 79.7% |

mDice; mean Dice, mIoU, mean Intersection over Union, MRM; medial rectus muscle.

**Table S8. Comparison of the performance of LRM segmentation for the Coronal image**

| **Target** | **Iteration** | **Model** | **mDice** | **mIoU** |
| --- | --- | --- | --- | --- |
|  |  | Proposed | 94.1% | 89.5% |
|  |  | HarDNet-MSEG | 83.9% | 74.8% |
|  |  | **Attention U-Net** | **94.1%** | **89.7%** |
|  |  | DeepLab V3+ | 91.4% | 84.6% |
|  |  | SegNet | 92.4% | 86.5% |
|  | 2 | **Proposed** | **94.9%** | **90.6%** |
|  |  | HarDNet-MSEG | 83.7% | 72.5% |
|  |  | Attention U-Net | 93.6% | 88.4% |
|  |  | DeepLab V3+ | 91.9% | 85.3% |
|  |  | SegNet | 93.0% | 87.3% |
|  | 3 | **Proposed** | **95.5%** | **91.8%** |
|  |  | HarDNet-MSEG | 87.4% | 78.2% |
|  |  | Attention U-Net | 94.8% | 90.8% |
|  |  | DeepLab V3+ | 83.9% | 78.0% |
|  |  | SegNet | 93.2% | 87.7% |
|  | 4 | **Proposed** | **95.8%** | **92.3%** |
|  |  | HarDNet-MSEG | 84.5% | 74.9% |
|  |  | Attention U-Net | 94.3% | 89.6% |
|  |  | DeepLab V3+ | 92.2% | 85.9% |
|  |  | SegNet | 93.1% | 87.6% |
|  | 5 | **Proposed** | **94.2%** | **89.7%** |
|  |  | HarDNet-MSEG | 82.0% | 71.8% |
|  |  | Attention U-Net | 93.7% | 88.7% |
|  |  | DeepLab V3+ | 90.3% | 82.6% |
|  |  | SegNet | 92.0% | 85.7% |
|  | 6 | **Proposed** | **94.9%** | **90.8%** |
|  |  | HarDNet-MSEG | 87.2% | 77.8% |
|  |  | Attention U-Net | 94.1% | 89.3% |
|  |  | DeepLab V3+ | 91.1% | 84.0% |
|  |  | SegNet | 92.8% | 87.2% |
|  | 7 | **Proposed** | **95.1%** | **91.3%** |
|  |  | HarDNet-MSEG | 89.0% | 80.8% |
|  |  | Attention U-Net | 94.3% | 90.0% |
|  |  | DeepLab V3+ | 90.8% | 83.7% |
|  |  | SegNet | 92.5% | 86.7% |
|  | 8 | **Proposed** | **94.7%** | **90.6%** |
|  |  | HarDNet-MSEG | 86.4% | 76.6% |
|  |  | Attention U-Net | 94.6% | 90.4% |
|  |  | DeepLab V3+ | 91.4% | 84.8% |
|  |  | SegNet | 93.1% | 87.5% |
|  | 9 | **Proposed** | **95.5%** | **91.8%** |
|  |  | HarDNet-MSEG | 90.2% | 82.5% |
|  |  | Attention U-Net | 94.7% | 90.3% |
|  |  | DeepLab V3+ | 92.0% | 85.6% |
|  |  | SegNet | 92.3% | 86.3% |
|  | 10 | **Proposed** | **94.8%** | **90.6%** |
|  |  | HarDNet-MSEG | 81.9% | 72.9% |
|  |  | Attention U-Net | 92.5% | 87.6% |
|  |  | DeepLab V3+ | 90.7% | 83.7% |
|  |  | SegNet | 91.4% | 84.8% |

mDice; mean Dice, mIoU, mean Intersection over Union, LRM; lateral rectus muscle.

**Table S9. Comparison of the performance of SRM segmentation for the Coronal image**

| **Target** | **Iteration** | **Model** | **mDice** | **mIoU** |
| --- | --- | --- | --- | --- |
| SRM | 1 | Proposed | 91.5% | 85.9% |
|  |  | HarDNet-MSEG | 86.2% | 76.3% |
|  |  | **Attention U-Net** | **92.6%** | **86.8%** |
|  |  | DeepLab V3+ | 90.9% | 83.8% |
|  |  | SegNet | 91.4% | 84.9% |
|  | 2 | **Proposed** | **93.0%** | **87.6%** |
|  |  | HarDNet-MSEG | 87.4% | 78.0% |
|  |  | Attention U-Net | 92.9% | **87.6%** |
|  |  | DeepLab V3+ | 90.5% | 83.3% |
|  |  | SegNet | 89.7% | 81.8% |
|  | 3 | **Proposed** | **94.6%** | **90.2%** |
|  |  | HarDNet-MSEG | 86.5% | 76.6% |
|  |  | Attention U-Net | 91.5% | 85.0% |
|  |  | DeepLab V3+ | 90.6% | 83.2% |
|  |  | SegNet | 92.2% | 85.9% |
|  | 4 | **Proposed** | **94.2%** | **89.5%** |
|  |  | HarDNet-MSEG | 83.1% | 72.3% |
|  |  | Attention U-Net | 93.7% | 88.6% |
|  |  | DeepLab V3+ | 91.5% | 84.7% |
|  |  | SegNet | 91.5% | 85.2% |
|  | 5 | **Proposed** | **92.3%** | **86.5%** |
|  |  | HarDNet-MSEG | 85.7% | 75.5% |
|  |  | Attention U-Net | 91.5% | 85.3% |
|  |  | DeepLab V3+ | 89.5% | 81.7% |
|  |  | SegNet | 89.5% | 82.2% |
|  | 6 | **Proposed** | **93.6%** | **88.5%** |
|  |  | HarDNet-MSEG | 83.3% | 72.3% |
|  |  | Attention U-Net | 92.9% | 87.3% |
|  |  | DeepLab V3+ | 89.9% | 82.2% |
|  |  | SegNet | 90.2% | 82.9% |
|  | 7 | **Proposed** | **93.2%** | **88.1%** |
|  |  | HarDNet-MSEG | 84.1% | 74.2% |
|  |  | Attention U-Net | 90.8% | 84.3% |
|  |  | DeepLab V3+ | 90.6% | 83.5% |
|  |  | SegNet | 91.4% | 85.0% |
|  | 8 | **Proposed** | **94.1%** | **89.3%** |
|  |  | HarDNet-MSEG | 82.9% | 71.3% |
|  |  | Attention U-Net | 91.2% | 84.3% |
|  |  | DeepLab V3+ | 90.7% | 83.4% |
|  |  | SegNet | 91.1% | 84.2% |
|  | 9 | **Proposed** | **93.3%** | **88.4%** |
|  |  | HarDNet-MSEG | 84.9% | 74.5% |
|  |  | Attention U-Net | 92.8% | 87.1% |
|  |  | DeepLab V3+ | 90.5% | 83.2% |
|  |  | SegNet | 90.3% | 83.0% |
|  | 10 | **Proposed** | **92.7%** | **87.6%** |
|  |  | HarDNet-MSEG | 87.0% | 77.9% |
|  |  | Attention U-Net | 91.8% | 86.2% |
|  |  | DeepLab V3+ | 88.3% | 80.5% |
|  |  | SegNet | 89.3% | 81.8% |

mDice; mean Dice, mIoU, mean Intersection over Union, SRM; superior rectus muscle.

**Table S10. Comparison of the performance of IRM segmentation for the Coronal image**

| **Target** | **Iteration** | **Model** | **mDice** | **mIoU** |
| --- | --- | --- | --- | --- |
| IRM | 1 | **Proposed** | **94.6%** | **90.2%** |
|  |  | HarDNet-MSEG | 83.4% | 72.1% |
|  |  | Attention U-Net | 93.6% | 88.8% |
|  |  | DeepLab V3+ | 89.9% | 82.0% |
|  |  | SegNet | 92.1% | 86.0% |
|  | 2 | **Proposed** | **94.4%** | **89.8%** |
|  |  | HarDNet-MSEG | 85.6% | 75.2% |
|  |  | Attention U-Net | 90.3% | 83.1% |
|  |  | DeepLab V3+ | 90.3% | 82.6% |
|  |  | SegNet | 89.1% | 81.4% |
|  | 3 | **Proposed** | **95.1%** | **91.1%** |
|  |  | HarDNet-MSEG | 88.3% | 79.3% |
|  |  | Attention U-Net | 94.7% | 90.3% |
|  |  | DeepLab V3+ | 90.3% | 82.8% |
|  |  | SegNet | 93.1% | 87.6% |
|  | 4 | **Proposed** | **95.1%** | **91.1%** |
|  |  | HarDNet-MSEG | 83.7% | 72.5% |
|  |  | Attention U-Net | 92.9% | 87.6% |
|  |  | DeepLab V3+ | 89.7% | 81.8% |
|  |  | SegNet | 92.8% | 87.1% |
|  | 5 | **Proposed** | **93.8%** | **89.1%** |
|  |  | HarDNet-MSEG | 83.7% | 72.6% |
|  |  | Attention U-Net | 93.7% | 88.8% |
|  |  | DeepLab V3+ | 89.7% | 81.7% |
|  |  | SegNet | 91.4% | 85.4% |
|  | 6 | **Proposed** | **94.6%** | **90.3%** |
|  |  | HarDNet-MSEG | 82.9% | 71.2% |
|  |  | Attention U-Net | 89.7% | 82.3% |
|  |  | DeepLab V3+ | 88.7% | 80.5% |
|  |  | SegNet | 90.9% | 84.2% |
|  | 7 | **Proposed** | **94.5%** | **90.0%** |
|  |  | HarDNet-MSEG | 81.4% | 71.2% |
|  |  | Attention U-Net | 93.9% | 89.1% |
|  |  | DeepLab V3+ | 89.6% | 81.5% |
|  |  | SegNet | 92.5% | 86.7% |
|  | 8 | **Proposed** | **94.5%** | **90.1%** |
|  |  | HarDNet-MSEG | 84.2% | 73.2% |
|  |  | Attention U-Net | 93.4% | 88.3% |
|  |  | DeepLab V3+ | 90.0% | 82.2% |
|  |  | SegNet | 92.2% | 86.1% |
|  | 9 | **Proposed** | **95.0%** | **90.9%** |
|  |  | HarDNet-MSEG | 84.7% | 73.9% |
|  |  | Attention U-Net | 91.7% | 85.5% |
|  |  | DeepLab V3+ | 88.3% | 79.9% |
|  |  | SegNet | 91.7% | 85.3% |
|  | 10 | **Proposed** | **93.0%** | **88.3%** |
|  |  | HarDNet-MSEG | 81.6% | 70.9% |
|  |  | Attention U-Net | 90.4% | 84.7% |
|  |  | DeepLab V3+ | 89.3% | 81.6% |
|  |  | SegNet | 88.6% | 82.0% |

mDice; mean Dice, mIoU, mean Intersection over Union, IRM; Inferior rectus muscle.
